# Self Assessment in Insects: Honeybee Queens Know Their Own Strength

**DOI:** 10.1371/journal.pone.0001412

**Published:** 2008-01-09

**Authors:** Vincent Dietemann, Huo-Qing Zheng, Colleen Hepburn, H. Randall Hepburn, Shui-Hua Jin, Robin M. Crewe, Sarah E. Radloff, Fu-Liang Hu, Christian W. W. Pirk

**Affiliations:** 1 Department of Zoology and Entomology, University of Pretoria, Pretoria, South Africa; 2 College of Animal Sciences, Zhejiang University, Hangzhou, China; 3 Department of Zoology and Entomology, Rhodes University, Grahamstown, South Africa; 4 Pinghu Breeding Apiary, Zhejiang, China; 5 Department of Statistics, Rhodes University, Grahamstown, South Africa; Indiana University, United States of America

## Abstract

Contests mediate access to reproductive opportunities in almost all species of animals. An important aspect of the evolution of contests is the reduction of the costs incurred during intra-specific encounters to a minimum. However, escalated fights are commonly lethal in some species like the honeybee, *Apis mellifera*. By experimentally reducing honeybee queens' fighting abilities, we demonstrate that they refrain from engaging in lethal contests that typically characterize their reproductive dominance behavior and coexist peacefully within a colony. This suggests that weak queens exploit an alternative reproductive strategy and provides an explanation for rare occurrences of queen cohabitation in nature. Our results further indicate that self-assessment, but not mutual assessment of fighting ability occurs prior to and during the agonistic encounters.

## Introduction

**Figure 1:**
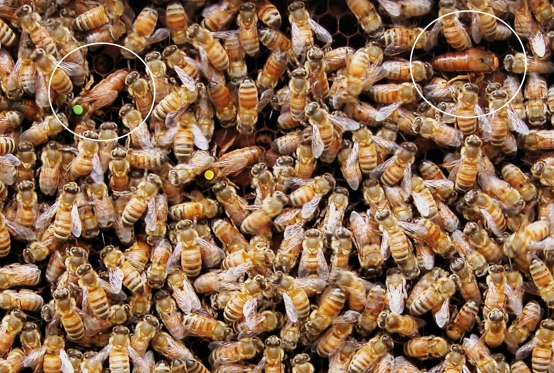
Three *A. m. ligustica* queens (circled) coexisting peacefully within a colony.

Most animals fight to gain access to mates and copulate [1], [2]. In many animal contests, fighting abilities of opponents are assessed directly during the physical interaction or indirectly when visual, auditory or chemical cues are correlated with fighting ability [1], [3]–[10]. These assessments enable an individual to gauge whether it should engage in a contest, and once engaged, to assess whether to persist if it is likely to win or to withdraw if the costs associated with losing become unacceptably high [4], [11], [12]. Escalation of fighting occasionally leads to fatalities [references in 2], [13], but lethal fights over resources are the general rule in a few species [14]. A textbook example of a species using lethal fights to settle a reproductive conflict is the honeybee, *Apis mellifera* [15]. Reproduction in honeybee colonies is achieved by colony fission. The mother queen leaves the nest with a group of workers to establish a new colony while several daughter queens are reared in the old nest. Rearing several replacement queens ensures that at least one will reach maturity and replace the departed queen. The first newly emerged queen kills her rivals within the cells in which they develop [16]. If several queens emerge simultaneously, they typically fight for reproductive supremacy until only one survives and takes over reproduction in the colony [17]. During the fights, the honeybee queens mount, grapple, and sting each other. Stinging is the usual cause of death and is only successful when queens have a good purchase with their mandibles on their opponents and can position themselves suitably [15], [18]. In China, beekeepers found a way to prevent queens from killing one another by ablating their mandibles. By forcing several queens to cohabit (Fig. 1), they create more productive colonies for commercial exploitation. In order to understand how manipulating the fighting ability of queens affects the social structure of honeybee colonies, we studied the effects of mandibular ablation on the strategic decisions of opponents during the fights.

## Results

**Figure 2:**
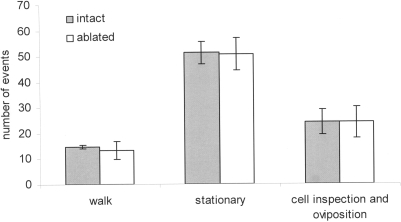
Levels of activity of intact queens that survived a contest (n = 3) and of ablated queens that cohabited peacefully in three colony. Activity levels are expressed as walking, being stationary, inspecting cells and ovipositing. A Mann-Whitney U test showed no significant difference between the groups.

When three queens with ablated mandibles were placed in an observation hive (n = 3), they did not kill each other and coexisted peacefully, making no attempts to fight. As a control, three intact queens were placed together in an observation hive (n = 3). Pairs of queens engaged in fights within 26.1±43.0 min (mean±s.d., range 1 to 113 min) after their introduction into the hive (Table 1). The majority fought on first contact (Table 2) and a single queen remained alive after 437.3±1001.0 min (mean±s.d., range 8–2480 min). Ablated queens were significantly more often in close proximity to each other than intact queens (expected vs. observed χ^2^ test, χ^2^ = 2082.9, df = 5, p<0.001; Table 2). Comparison of movement patterns, oviposition rate and frequency of cell inspection by ablated and intact queens showed no significant differences between the two groups (Mann-Whitney U test: U_walking_ = 7.5, p_walking_ = 0.50, NS; U_stationary_ = 10.0, p_stationary_ = 0.91, NS; U_oviposition/cell inspection_ = 9.5, p_oviposition/cell inspection_ = 0.82, NS; Fig. 2). Throughout the study, no aggression between queens and workers was observed.

**Table 1:** Delay in minutes between introduction of the queens into the observation hives and the start of the fights.

	ablated queens			intact queens			2 ablated/1 intact queens		
colony	1	2	3	4	5	6	7	8	9
queen 1 vs. 2	-	-	-	1	6	18	20	77	14
queen 2 vs. 3	-	-	-	6	12	113	24	132	18
mean+s.d.	-			26.1±43.0			46.0±44.4		

– indicates that no fighting occurred. The delay between introduction and fighting for the second pair of queens that interacted in a colony is calculated by subtracting the duration of the fight involving the first pair of queens that fought in this colony.

**Table 2:** Proximity of queens in the observation hives.

group of	ablated queens			intact queens		
colony	1	2	3	4	5	6
queen 1 vs. 2	0*	11	4	1	1	2
queen 2 vs. 3	2	9	12	1	2	1
queen 1 vs. 3	0*	0	19	-	-	-
mean+s.d.	8.1±6.6			1.3±0.5		

Proximity is expressed in frequency of queen presence in the same 6×6 cm square of the grid covering the combs during a five-day period. Fights never occurred among queens deprived of one of their mandibles (ablated queens). – indicates that queens were never observed in the same square since only one queen remains alive after two fights. * indicates queens that died due to human manipulation before completion of the observations and were excluded from the calculations.

In order to control for the effect of mandibular ablation on queen recognition and release of fighting behavior [19], we placed two ablated queens together with one intact queen in an observation hive (n = 3 replicates). Fighting occurred after 46.0±44.4 min, significantly later than between intact queens (expected vs. observed χ^2^ test, χ^2^ = 167.2, df = 1, p<0.001; Table 1). In these mixed groups of ablated and intact queens, the latter were always involved in the first two fights taking place between the three queens (there are 3 combinations of queen pairs, but all queens took part in a contest after 2 fighting pairs interacted). The intact queen initiated at least 67% of these fights (four out of six fights in the 3 replicates; the identity of the queen initiating the remaining two fights could not be determined). After the ablated queens were attacked by an intact queen, the ablated queen also displayed aggressive behavior and engaged in fights with one another and against intact queens.

## Discussion

Honeybee queens refrain from fighting to death when one of their mandibles is missing. An explanation of the fact that these ablated queens do not fight is that their general activity level could be depressed following mandible ablation. We demonstrated, based on movement pattern and oviposition and cell inspection rates, that this was not the case. Ablated queens displayed the same activity level as intact queens that survived a contest. In addition, ablated queens were often observed in close proximity, indicating that the lack of fighting amongst them could not be attributed to spatial separation. The longer delay in fight initiation in mixed groups composed of ablated and intact queen in comparison to groups composed of intact queens only is likely due to the poor initial motivation to fight by the ablated queens. This idea is supported by their peaceful behavior in the three experimental groups composed exclusively of ablated individuals. However, subjection to attack triggered the aggressiveness of ablated queens both towards intact queens and towards other ablated queens. Mandibular ablation does therefore not alter queen recognition or the release of fighting behavior, and does not inactivate the queens' fighting capacity, but it influences their decision to engage in a contest.

Strategic decisions of an individual during contests are influenced by internal and external factors [1]. Models based on game theory have considered the possibility that contestants gauge their own fighting ability and not that of their opponent [20]–[22], and results from some species suggest that contestants determine their fighting strategy solely based on self-assessment [23]–[26], an internal factor. Self-assessment is adaptive when mutual assessment is costly [24] and this is indeed the case in honeybees since there is a high probability that once engaged in a fight, it results in the death of one contestant [15]. Self-assessment can occur during a contest, when an individual will decide to retreat when the costs inflicted by the opponent reaches a certain threshold [1], [24]. Alternatively, self-assessment can be based on a benchmark that allows the determination of an individual's strength compared to the population's average [23], [27]. Honeybee queens in our experiment had no prior fighting experience on which to base their strategic decisions and refrained from engaging in a fight. Indeed, in accordance with standard beekeeping practices, the queens used in this experiment were reared *en masse* and reintroduced individually into their colony of origin before the start of our experiments. These queens did not establish themselves as reproductives by taking part in, and winning, a fight. Therefore, they had no prior knowledge of their own strength in comparison to other queens or of how they would perform during a fight. Unless they had an inherent knowledge of population average fighting ability, honeybee queens seem to rely solely on an assessment of their own absolute fighting ability [28] to make their decision prior to any physical contest. Thus, despite having no experience of fighting, it is likely that the queens deprived of one mandible recognized their poor fighting ability. This self-assessment could occur through a physiological reaction to mandibular ablation, through the absence of mechanical stimuli arising from contact by both mandibles upon closure or during interactions with workers.

Another possibility to explain the peaceful behavior of ablated queens is that they obtained information about the fighting ability of their opponents in an indirect and remote manner. This mutual assessment must occur before physical contact since when queens fought, they usually did so on their first encounter. As honeybees live in darkness in the hives, it is unlikely that they use visual cues to assess each others. However, honeybee queens are known to communicate by sound [15] and pheromones [29], thus it is possible that certain components of these cues [30], [31] may be used as an indicator of fighting ability. Honest signaling of fighting ability by queens can be ruled out based on the peaceful behavior of queens in the ablated groups. Under an honest signaling mechanism, queens in ablated groups should have identified opponents of equal fighting ability and engaged in contests (since they all possessed similar handicaps and had equal probabilities of a winning an encounter). Alternatively, if these queens signaled dishonestly, they should advertise a strong fighting ability, but refrain from engaging in a fight due to their weak fighting ability. Absence of fighting was a characteristic of our observations, intimating that dishonest communication by queens provided information on fighting ability and that mutual assessment occurred prior to physical contact. However, we consider dishonest signaling an unlikely scenario since fighting to the death is common in honeybee colonies (44% of the queens are killed before they emerge and 36% of the newly emerged queens die during a duel [16]) and cheaters, queens producing dishonest signals, would be discovered with relatively high frequency [32]–[34], making deception evolutionary unstable in this system. Mutual assessment, an external factor is therefore unlikely to occur during contest between honeybee queens. Further external factors [1] do not seem to influence their decisions: the value and knowledge of the contested resource was the same for all contestants, their age and prior experience were the same and the experimental groups being newly created with workers unrelated to them, none of the contestants had an ownership or kinship advantage [1]. Their avoidance of confrontation therefore seems to be based solely on internal factors.

The fact that no death occurred in our groups with ablated queens (ablated or mixed groups) suggests that mandibular ablation prevents the killing of opponents. Ablated queens in our experiment or weak queens in nature could refrain from fighting since self-assessment could show them they have low probability of winning. It is likely that weak queens were selected to avoid fights since probability of dying is high when their ability to hold onto their opponent and sting (i.e. to attack and kill an opponent) is reduced. Indeed, for a weak queen that does not have information about its opponent's fighting ability, the best strategy for a higher chance of surviving is not to initiate a contest. In contrast, intact or strong queens detect the presence of other queens and engage into fights. Although they have the potential to kill their opponent in mixed groups, they stopped fighting before killing the ablated queens. Self-assessment during the fight could show the strong individual that a weak opponent does not represent a threat to its life and could let it live, provided that its presence is not detrimental to the strong individual's fitness. Multiple queen colonies in China are used to increase brood production, corroborating the idea that the presence of ablated queens is not detrimental, but benefits the colony through a faster build-up. Additionally, under natural conditions newly produced queens are related and therefore the strong individuals might acquire inclusive fitness by leaving the queens live and reproduce.

Potential assessment mechanisms of the opponents' fighting ability during a contest are described in several game theory models [20], [22], [35]. If these models explain the functional significance of strategic decisions during animal fights, the decision processes that underlie the behavior of the contestants are poorly known, with few exceptions [6], [9], [36]. Since most studies focus on identifying predictors of fight outcome (on which contestants can base their strategic decisions), fighting has to take place for the experimenter to determine the outcome. Escape by opponents is generally constrained by experimental conditions that force the individuals into a confrontation [37], [38], which decreases the options available to the contestants and increases the frequency of escalation. Moreover, conflict avoidance is rarely quantified [6], [8], [37]–[41 for exceptions]. These apparent biases in experimental design result in misinterpretation of the strategic decisions made by the contestants and distort our understanding of animal conflicts. As a result, the effect of an individual's own fighting ability or handicaps on motivation to fight is poorly known. In the case described here, avoidance was possible since three ablated queens placed together in a hive did not fight. As a result, the absence of killing in some of our experimental groups suggests that there are previously underestimated alternatives to lethal fights for queens and could explain the variability in the events observed during swarming processes [16]. This idea is confirmed by the fact that our observations correspond to the prediction of game-theory modeling that mathematically illustrates that when a highly valuable resource is contested and the alternative to winning the fight is poor, individuals should fight to the death irrespective of their own or their opponents' fighting ability [14]. Queen survival in our experiments is easily explained if the alternative to losing a fight, i.e. the future fitness of a contest loser, is not poor. Since they cannot found colonies independently and need the help of workers to build a new nest, honeybee queens have the alternative opportunity to leave with a secondary swarm when the colony remains large enough after departure of the prime swarm and when conditions are favorable [15], [17]. Although this option is less risky than engaging into a lethal fight, it is not without dangers since the probability of founding a new colony with a secondary swarm is low [16]. In such a situation, the interests of both queens coincide: the strong queen inherits the more valuable existing nest and obtains indirect fitness if the weak queen successfully establishes a new colony. Fighting to the death would therefore be unnecessary to settle the dispute and costs of injuries would be minimized. The weak queens can thus trade off certain death against the prospect of establishing a new colony. However, irrespective of the queens' advantages, the final decision about swarming might belong to the workers, since they can delay queen emergence, initiate swarming, and force queens to leave with the swarms [30].

Our results show, that during contests for reproductive monopoly, honeybee queens make strategic decisions without prior experience [37], based solely on the assessment of their own absolute fighting ability [24]. That lethal fighting represents the rule and coexistence the exception in nature can be explained by the life history of honeybees. Under natural conditions, newly-emerged queens are sisters that have been raised in the same environment; it is therefore likely that they have similar fighting abilities and that neither of them is willing to settle for a less rewarding option than colony take over, leading to lethal contests. Nevertheless, queens deprived of a weapon refrain from fighting, suggesting that they can avoid lethal fights and adopt an alternative reproductive strategy. Therefore, contrary to expectation arising from the lethal nature of the fighting [14] between honeybee queens, there are conditions where alternative strategies are adaptive. Studying reproductive competition and the diversity of its outcomes can help us understand the complexity of conflict resolution within insect societies.

## Materials and Methods

### Experimental setup

The experiments were carried out in May 2005 and 2006 in Hangzhou, China with colonies of the ‘Italian honeybee’, *Apis mellifera ligustica*. The queens used in this experiment were marked individually with color tags and were unrelated. In order to decrease the fighting ability of some individuals, two thirds of one of their mandibles was cut off with scissors. The operated queens were replaced in their colony of origin for one week to heal before they were used in the experiment. At the start of the experiment, a random sample of 2500 one to two days-old workers were placed in two-frame observation hives (48.8×49×5.6 cm) in free foraging conditions. These workers originated in 20 colonies that were unrelated to those that provided the queens to exclude potential nepotism by workers that could influence queen duels. In each these observation hive (n = 3 for each group), we placed three ablated queens or two ablated queens and a sham-operated queen (that possessed both mandibles, thereafter designated as intact queens) or three intact queens.

### Behavioral observations

The behavior of each queen in a hive was monitored by a different observer. We noted the identity of the queens initiating the fight and the subsequent outcome. In one of the replicates in which three queens had ablated mandibles, one queen died during the night of the second to the third day of experiment and another queen died the morning of the fourth day. They had not engaged in fights, and the cause of their death was probably human manipulation at their introduction in the hive. Fights in colonies in which all queens were intact were monitored and recorded continuously from queen introduction until death of all queens but one. Colonies in which ablated queens were introduced or in which a single queen remained were observed for 10 min bouts every hour between 9:00 and 17:00 during five days. Colonies hosting more than one queen were observed continuously but data was only recorded as mentioned above or when fighting occurred. Since mandible ablation could affect the queens' general activity levels and movements, and hence their ability to find and attack rivals, we monitored whether the queens were stationary, walking, inspecting cells or ovipositing between 9:00 and 17:00 during non-continuous observations, the day before the last observations were carried out. We also noted the distance between queens to determine whether they were able to interact. For this, the position of the queens on a grid of 7 by 12 cells of 6×6 cm was noted for every observation made. The number of occurrences of the event ‘queens present in the same square’ were cumulated for each pair of queens until the end of the observation period or until queens fought.

### Statistical analysis

An observed versus expected χ^2^ test was used to compare how frequently queens were observed in close proximity (i.e. in the same square of the grid) in the different treatments. The expected frequency is the mean proximity of intact queens before the first fight. The observed frequency is the mean proximity of ablated queens during the 5 days experiment. The same statistics were used to compare the delay before fighting occurred in the different treatments. The expected frequencies are the proportions of delays higher and lower than the median delay between introduction and fighting between intact queens. The observed frequencies are the proportions of delays higher and lower than the median delay of fighting between ablated and intact queens and amongst ablated queens. A Mann-Whitney U test was used to compare the levels of activity (walking, being stationary, inspecting cells and ovipositing) of intact queens that survived lethal contests to that of ablated queens that coexisted peacefully. Statistica 7.1 ® was used for the analyses.
